# SAD-SNN: Spatial-Activation Distillation for High-Performance Spiking Neural Networks

**DOI:** 10.3390/s26154877

**Published:** 2026-08-02

**Authors:** Chongxiao Qu, Qian Zhang, Chenxiao Dou, Xiaohu Li, Xinyu Chen, Zhenyu Zhao, Baoqing Zeng, Xinwei Yao

**Affiliations:** 1 School of Electronic Science and Engineering, University of Electronic Science and Technology of China, Chengdu 611731, China; 2 China Nanhu Academy of Electronics and Information Technology, Jiaxing 314002, China; 3 Academy of Advanced Interdisciplinary Science and Technology, Zhejiang University of Technology, Hangzhou 310014, China

**Keywords:** spiking neural networks, knowledge distillation, image classification, electromagnetic signal detection

## Abstract

A Spiking Neural Network (SNN) is a kind of brain-inspired and event-driven network, which is becoming a promising energy-efficient alternative to Artificial Neural Networks (ANNs). In recent years, SNN methods have been successfully applied in the fields of electromagnetic signal processing and image signal processing, particularly in application scenarios that require low energy consumption. However, the performance of SNNs by direct training is far from satisfactory. In this paper, we study a novel learning method named SAD-SNN (Spatial-Activation Distillation for Spiking Neural Networks), which utilizes the ANN model to guide the SNN model learning. Unlike prior works that rely on element-wise feature alignment approaches, SAD-SNN aligns spatial-activation maps at different resolutions of the teacher and student networks. Specifically, we introduce a direct alignment approach, which defines a spatial-activation loss and normalizes the representation vectors of ANN and SNN, to alleviate the unexpected precision loss. This enables the knowledge of teacher ANNs to be effectively transferred to train student SNNs. On three image classification datasets, our proposed SAD-SNN outperforms other SNN training methods no matter whether homogeneous or heterogeneous teacher ANNs are used. Furthermore, we apply SAD-SNN to the electromagnetic signal detection task, demonstrating strong generalization ability and superior performance. In conclusion, the experimental results on various tasks and SNN architectures demonstrate that our method is a general and effective solution that significantly improves the learning of student SNNs with only two time steps.

## 1. Introduction

A Spiking Neural Network (SNN) is a kind of biologically plausible neural network based on dynamic characteristics of biological neurons [1,2]. Previous research has demonstrated the potential of SNNs in achieving energy savings while enabling fast inference [3]. Recently, many SNN methods [4,5,6] have exhibited significant low-power advantages in tasks of image signal processing and electromagnetic signal processing. However, the accuracy performance of those direct-trained SNNs is not comparable to that of ANNs. Although surrogate gradient methods [7,8] are proposed to improve SNN training, the results are often accompanied by lower accuracy and slower convergence rates than those of ANNs.

Knowledge distillation (KD) [9] has recently gained attention as an effective strategy for improving SNN training. By treating pre-trained high-performance ANNs as teachers and target SNNs as students, KD methods guide SNN learning by transferring semantic knowledge from ANNs. Typically, most existing feature-based approaches focus on element-wise alignment [10,11] of intermediate features between teacher ANNs and student SNNs. However, these methods often overlook the representation gap: ANNs generate continuous floating-point features with dense information distribution, whereas SNNs produce discrete binary spikes characterized by sparsity and temporal dynamics. This fundamental distributional mismatch restricts knowledge transfer and results in unexpected precision loss during distillation.

**Figure 1:**
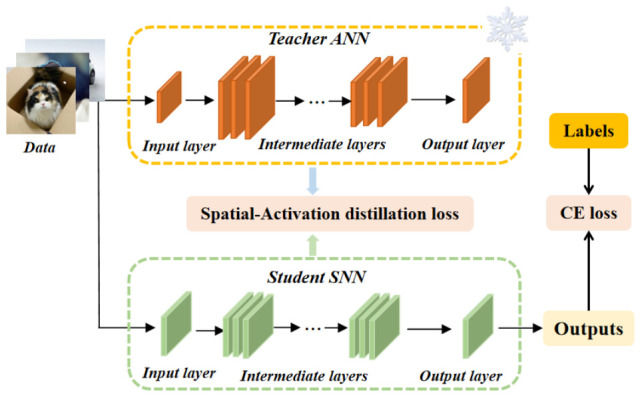
Overview of training SNN with Spatial-Activation Distillation method. The snow symbol indicates that the parameters of the teacher ANN are frozen.

Considering the above challenges, in this paper, we propose a novel learning method named SAD-SNN (Spatial-Activation Distillation for Spiking Neural Networks), as illustrated in Figure 1, which leverages feature-based knowledge distillation to transfer comprehensive supervisory information from a pre-trained ANN teacher to an SNN student. To address the limitations of prior element-wise feature alignment methods, SAD-SNN aligns spatial-activation maps across different spatial resolutions between the teacher and student networks. Specifically, we introduce a spatial-activation loss with a normalization operation to align the representation vectors of ANN and SNN at each corresponding layer, enabling effective knowledge transfer across heterogeneous networks. Unlike prior methods that rely on high-dimensional feature transfer or stochastic perturbation, our approach is simple, parameter-free, and enables effective cross-architecture knowledge transfer with only two time steps.

To summarize, our contributions are threefold:

- We propose SAD-SNN, a feature-based distillation framework that transfers knowledge from ANNs to SNNs via spatial-activation alignment across different resolutions.
- We introduce a spatial-activation loss with normalization to bridge the distribution gap between continuous ANN features and sparse SNN spikes, without introducing additional trainable parameters.
- We benchmark our method on image classification and electromagnetic signal detection tasks. Experimental results show that the proposed SAD-SNN outperforms other SNN training methods with only two time steps, demonstrating its reliability and validity.

## 2. Related Work

### 2.1. SNN Training and Applications

Current deep SNN training methods can be broadly divided into two main categories: indirect supervised learning, typified by ANN-to-SNN conversion [12], and direct supervised learning, typified by spatio-temporal backpropagation [7,8].

ANN-to-SNN conversion pre-trains a source ANN and then converts it into an SNN by replacing the rectified linear unit (ReLU) activation function in artificial neurons with a spiking neuron model [13], while sharing the trained weights and without further training of the SNN. Despite its effectiveness in generating deep SNNs, this approach often overlooks the rich temporal dynamic characteristics inherent to SNNs. Moreover, it requires longer time steps to match the accuracy of pre-trained ANNs [14], which increases SNN latency and limits practical applicability.

Directly trained SNNs address the non-differentiability of spikes by introducing surrogate gradient methods. These methods implement spatio-temporal backpropagation by approximating the gradient with smooth functions [15,16]. The vanishing gradient problem is also unavoidable in SNN training, and some research has proposed novel normalization techniques to alleviate this issue [17]. It is worth noting that direct supervised learning requires substantially fewer time steps compared to ANN-to-SNN conversion.

The advancement of these training methods has facilitated the deployment of SNNs in various application scenarios [18]. In image signal processing, SNNs have achieved promising performance on public benchmarks [19] with significantly lower energy consumption compared to ANNs [20]. For electromagnetic signal processing, SNNs have shown promising results in automatic modulation recognition [5] and radar emitter recognition [21], leveraging their temporal dynamics and event-driven computation to achieve robust performance even under low signal-to-noise ratio conditions. These application-driven studies motivate the development of efficient training methods to further bridge the performance gap between ANNs and SNNs in practical scenarios.

### 2.2. Knowledge Distillation for SNNs

Knowledge distillation (KD) [22] is a model compression technique that transfers knowledge from a large teacher model (or an ensemble of models) to a smaller student model, providing richer supervisory signals for the student’s training.

Recently, many works have applied knowledge distillation to the Spiking Neural Network (SNN) domain [23,24]. For instance, ref. [10] leverages the output responses of teacher ANNs to enhance student SNNs by aligning output logits and intermediate features. Subsequently, ref. [25] proposes a temporal separation strategy with entropy regularization, which performs distillation on logits at individual time steps rather than merely on aggregated outputs, thereby better exploiting the spatio-temporal properties of SNNs. To address the precision gap between continuous ANN features and discrete SNN spikes, ref. [11] introduces blurred knowledge distillation, which applies random blurring masks to SNN features before mimicking ANN features. However, this stochastic operation may obscure fine-grained spatial information. Additionally, ref. [26] proposes a biologically inspired reverse knowledge distillation approach that transfers structural pattern learning from ANNs to SNNs through reversed distillation pathways, though it requires a multi-stage training pipeline.

In this work, we propose SAD-SNN, a feature-based distillation approach for ANN-SNN training. Prior methods mainly focus on how to align, using element-wise feature matching, stochastic perturbation, or reversed distillation pathways. We focus on what to align: spatial-activation maps across different resolutions, bridging the representation gap between continuous ANN features and discrete SNN spikes, without introducing any trainable parameters.

## 3. Preliminary

**Leaky Intergrate-and-Fire Model.** Unlike traditional ANNs, SNNs use binary spike trains to transmit information. In this paper, we use the Leaky Integrate-and-Fire (LIF) neuron [7] as the basic neuron model of student SNNs. When the membrane potential exceeds a specific threshold, the neuron fires a spike and then the membrane potential is reset to zero. The LIF model is defined as

(1) $$u^{t,l+1}=\tau u^{t-1,l+1}\left(1-o^{t-1,l+1}\right)+x^{t,l}$$

(2) $$o^{t,l+1}=\left\{\begin{array}{cc} 1\; & \mathrm{if}\;u^{t,l+1}>\theta^{l+1}\\ 0\; & \mathrm{otherwise} \end{array}\right.$$

where $u^{t,l}$, $x^{t,l}$ and $o^{t,l}$ are the membrane potential, input current and spike output of the neuron in the *l*-th layer at time *t*. τ represents the membrane time constant, describing how fast the membrane decays. $\theta^{l}$ is the threshold in the *l*-th layer. This LIF model enables forward and backward propagation to be implemented on both spatial and temporal dimensions.

**Integer Leaky Integrate-and-Fire Model.** The I-LIF neuron [27] shares the same membrane potential dynamics as the standard LIF, but differs in the output generation. Instead of emitting binary spikes, I-LIF produces integer-valued outputs:

(3) $$o^{t,l+1}=\mathrm{Clip}\left(\mathrm{round}\left(u^{t,l+1}\right),0,D\right)$$

where *D* is a hyperparameter controlling the maximum integer value. When D=4, the per-time step capacity expands from 1 bit to approximately 2.32 bits, enabling strong performance even at T=1. This observation highlights that the performance gap between ANNs and SNNs is fundamentally rooted in representational capacity.

**Notations.** ANNs are effective at learning hierarchical feature representations with increasing levels of abstraction [28]. Therefore, not only the last layer [22] but also the intermediate layers [29] can be utilized as the knowledge sources to supervise the training of student SNNs. Such layer outputs are so called feature maps [30]. Denote a teacher ANN as T and a student SNN as S. For an input mini-batch, the feature map of T at layer *l* is $A_{T}^{l}$, and that of S at layer $l^{\prime }$ is $A_{S}^{l^{\prime }}$ (with an additional temporal dimension *T*). We use I to denote the set of aligned layer pairs $\left(l,l^{\prime }\right)$. For ease of reference, we summarize the key notations in Table 1.

## 4. Methodology

In this section, we first give an introduction to the spatial-activation knowledge. Then, based on the knowledge definition, a spatial-activation loss function is proposed. Finally, the overall training process is fully described.

### 4.1. Spatial-Activation Knowledge

In this paper, we adopt the spatial-activation maps inspired by [31] to align the feature representations of the teacher ANN and the student SNN by measuring their spatial similarity. As SNNs have an additional dimension of time, for generality, we take the strategy of average pooling [10] to get rid of the time dimension. After the step of average pooling over the time dimension, SNN’s feature map will transfer from $A_{S}^{l^{\prime }}\in R^{B\times T\times C^{\prime }\times H^{\prime }\times W^{\prime }}$ to $A_{S^{\prime }}^{l^{\prime }}\in R^{B\times C^{\prime }\times H^{\prime }\times W^{\prime }}$, which has the same dimension as ANN’s feature map $A_{T}^{l}\in R^{B\times C\times H\times W}$.

**Figure 2:**
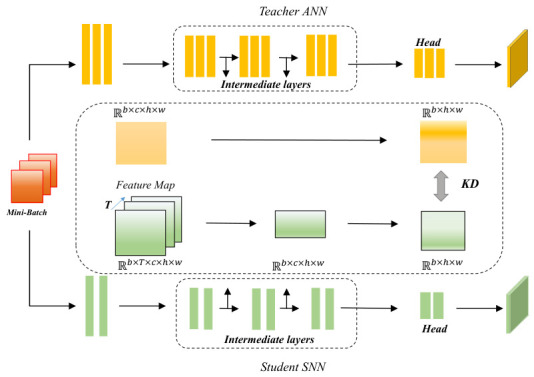
Illustration of feature-based distillation for SNNs and use of average pooling to get rid of the time dimension of SNN’s feature maps and calculating the spatial-activation maps of ANN and SNN.

As shown in Figure 2, for the ease of transfer learning, we derive spatial-activation maps from feature maps to represent the knowledge of network layers. The student SNN is encouraged to produce similar spatial-activation maps as the teacher ANN, which can be formulated as follows

(4) $$S_{T}^{l}=\frac{1}{C}\sum_{i=1}^{C}|A_{T[:,i,:,:]}^{l}{|}^{2};\;S_{S^{\prime }}^{l^{\prime }}=\frac{1}{C^{\prime }}\sum_{i=1}^{C^{\prime }}|A_{S^{\prime }\left[:,i,:,:\right]}^{l^{\prime }}{|}^{2}$$

where $S_{T}^{l}\in R^{B\times H\times W}$ and $S_{S^{\prime }}^{l^{\prime }}\in R^{B\times H\times W}$ denote the $\left(l,l^{\prime }\right)$ layer pair of teacher and student spatial-activation maps, respectively. The notation [:,i,:,:] denotes the *i*th channel in the matrix.

### 4.2. Spatial-Activation Distillation

According to the definition of spatial-activation maps above, there exists a potential problem when computing the similarities in SNNs. In the feature maps of SNNs, as the values of most features are often 0, the similarity vectors computed in SNNs may be very sparse, making it hard to match those dense vectors of ANNs.

Considering this matching problem, we define the spatial-activation loss as

(5) $$L_{distill\_SAD}\left(S_{T},S_{S^{\prime }}\right)=\frac{1}{B}\sum_{i=1}^{B}\sum_{(l,l^{\prime })\in I}||\mathrm{norm}\left(S_{S^{\prime }\left[i,:,:\right]}^{l^{\prime }}\right)-\mathrm{norm}\left(S_{T[i,:,:]}^{l}\right){\left|\right|}_{2}.$$

where the notation [i,:,:] denotes the *i*th data sample, the set I contains the layer pairs $\left(l,l^{\prime }\right)$ that are aligned (e.g., end of each stage or each block). The function norm(·) denotes instance-wise $L_{2}$ normalization, i.e.,  $\mathrm{norm}\left(v\right)=v/{\left||v|\right|}_{2}$, applied to each sample’s spatial-activation vector independently. This normalization unifies the value scales of ANN and SNN similarity vectors to the range [0,1], alleviating the precision gap caused by their different activation distributions.

By combining the Spatial-Activation Distillation loss with the original task loss, the total loss $L_{total}$ is given as follows.

(6) $$L_{total}=L_{task}+\alpha *L_{distill\_SAD}$$

where $L_{task}$ denotes the task-specific loss between the predictions and the ground truth, and α is a coefficient used to balance the two losses.

### 4.3. Training

In this section, we provide a comprehensive description of our proposed *SAD-SNN* for training SNNs.

**Training teacher ANNs.** We begin by training ANNs as pre-trained teacher models. From these teacher networks, we extract the outputs from their intermediate layers as feature maps and calculate the spatial-activation maps that serve as guiding signals during the distillation process for training SNNs.

**Training student SNNs.** SAD-SNN guides the training of a student SNN by incorporating an additional distillation loss. In the forward propagation, the same set of samples are both input to the teacher ANN and the student SNN. The student SNN encodes its outputs into spike firing rates, which serve as its feature representations. The pre-trained teacher ANN provides its final outputs or intermediate feature maps as the target knowledge representation. The total loss is then formulated based on the hidden knowledge representation, as defined in (5). In the error backpropagation, the classical backpropagation algorithm cannot be directly applied due to the non-differentiable nature of the spike activity function in (2). To address this issue, most previous works exploit surrogate gradients for the spatio-temporal backpropagation algorithms [7,32]. In this study, we employ a threshold-dependent batch normalization method [33] to train SNNs. This method uses a rectangular function [7] to approximate the derivative of spiking activity, enabling direct training of SNNs from shallow to deep architectures. The pseudocode for the overall training process of SAD-SNN is summarized in Algorithm 1.
**Algorithm 1** Training Algorithm of SAD-SNN**Input:** Dataset D. **Model:** Teacher ANN model $M_{\mathrm{ANN}}\left(x;W_{\mathrm{ANN}}\right)$ with pretrained weights $W_{\mathrm{ANN}}$; Student SNN model $M_{\mathrm{SNN}}\left(x;W_{\mathrm{SNN}}\right)$ with initial weights $W_{\mathrm{SNN}}$. **Parameter:** Time steps *T*; Layer alignment set I; Distillation loss weight α; Learning rate η; Training epoch *E*. **Output:** Trained student SNN model $M_{\mathrm{SNN}}\left(x;W_{\mathrm{SNN}}\right)$. 1: **for** 
epoch=1 
**to** 
*E* 
**do** 2:     **for** *x*, *y* **in** D **do** 3:         $A_{T}=M_{\mathrm{ANN}}\left(x\right)$; ▹ forward propagation 4:         $A_{S},\hat{y}=M_{\mathrm{SNN}}\left(x\right)$; 5:         $A_{S^{\prime }}={\mathrm{AvgPool}}_{T}\left(A_{S}\right)$; ▹ calculate spatial-activation maps 6:         calculate $S_{T}$ and $S_{S^{\prime }}$ based on $A_{T}$ and $A_{S^{\prime }}$ using (4); 7:         calculate $L_{distill\_SAD}$ based on $S_{T}$ and $S_{S^{\prime }}$ 8:                               using layer pairs $(l,l^{\prime })\in I$ via (5); ▹ calculate $L_{distill\_SAD}$ 9:         calculate task-specific loss based on *y* and $\hat{y}$; ▹ calculate $L_{task}$ 10:          calculate $L_{total}$ based on $L_{task}$ and $L_{distill\_SAD}$ using (6); ▹ calculate $L_{total}$ 11:          calculate the gradients $\frac{\partial L_{\mathrm{total}}}{\partial W_{SNN}}$; ▹ backward propagation 12:          update $W_{SNN}$; ▹ update weights 13:      **end for** 14:  **end for**


## 5. Experiment

### 5.1. Experiment Overview

In this section, we systematically present our experimental approach, which thoroughly evaluates the proposed SAD-SNN framework across different tasks, datasets, and architectures. We first focus on the widely adopted image classification task, verifying the effectiveness of our method on multiple datasets, including CIFAR10, CIFAR100, and Tiny ImageNet, using ResNet and PyramidNet architectures. Subsequently, we extend our framework to tackle the challenging electromagnetic signal detection task in communication reconnaissance, where we adopt YOLOv5 Medium and YOLOv8 Medium as teacher ANNs and SpikeYOLO as the student, evaluated on the signal detection dataset. This comprehensive experimentation demonstrates the strong generalization ability of SAD-SNN, which can be effectively applied to both classification and detection tasks across fundamentally different data modalities.

### 5.2. Classification

#### 5.2.1. Implementation Details for Classification

**Datasets.** We evaluated our proposed methods on three datasets: CIFAR10 [34], CIFAR100 [35], and Tiny ImageNet. CIFAR10 and CIFAR100 contain 60 k RGB images of size 32×32×3, with 10 and 100 categories, respectively. Each dataset is split into 50,000 training samples and 10,000 testing samples. Tiny ImageNet contains 110 k RGB images of size 64×64×3 across 200 classes, and is a subset of ILSVRC-2012. For this dataset, each class contains 500 training samples and 50 testing samples.

**Backbone Architectures.** We employed five representative architectures as teacher ANNs to evaluate the transfer performance of SAD-SNN: ResNet19 [36], ResNet34, PyramidNet110 (PyrNet110) [37], PyramidNet50 (PyrNet50), and WideResNet28 (WRN28) [38]; and we employed ResNet19 [17] and PyramidNet50 (PyrNet50) as student SNNs.

**Feature Alignment.** For SAD-SNN, we align the last three feature layers of the teacher ANN and the student SNN, respectively. Specifically, for each network, we identify the outputs of its final three feature extraction stages (or convolutional blocks) before the classification head. These corresponding layer pairs are directly aligned regardless of architectural differences or channel dimensions.

All experiments were conducted on a single NVIDIA A100 GPU with 80 GB memory using a fixed random seed of 42. For teacher ANNs, the number of epochs was set to 100, 200, and 200 for CIFAR10, CIFAR100, and Tiny ImageNet, respectively. The batch sizes were 64 for CIFAR10/100 and 128 for Tiny ImageNet. We adopted the SGD optimizer with an initial learning rate of 0.025, which decayed to 0.0025 at the halfway point of training. For PyrNet110 and PyrNet50, the widening factor $\alpha_{w}$ and the output feature dimension were set to 270 and 286, respectively. During SNN training, the number of epochs was set to 100, 200, and 200 for CIFAR10, CIFAR100, and Tiny ImageNet, respectively, and the batch size was set to 64. The hyperparameter α of our proposed method was set to 1000 for all datasets. The time step was set to 2. We adopted the Adam optimizer with an initial learning rate of 0.001, which decayed to 0.0001 at the halfway point of training. For PyrNet50, the widening factor $\alpha_{w}$ and the output feature dimension were set to 270 and 286.

#### 5.2.2. Performance Comparison on Benchmarks

**Figure 3:**
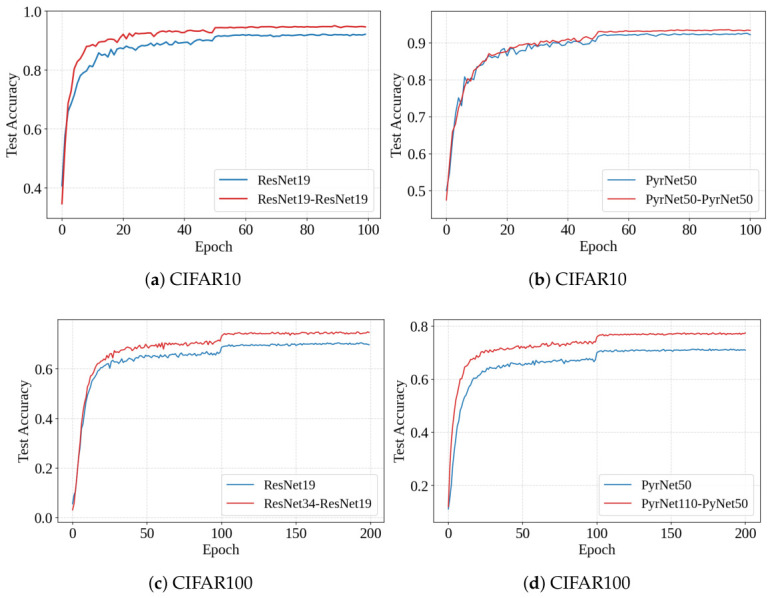
Test accuracy curves of directly trained SNNs and student SNNs on CIFAR datasets.

**Learning from the homogeneous ANNs.** We selected ResNet19 and PyrNet50 as student SNNs to evaluate the performance of SAD-SNN when transferring knowledge from homogeneous ANNs. This includes scenarios where the student and teacher networks share the same depth or have the same block structure but different depths. We compared our student SNNs with current leading SNN training methods. Experimental results on two benchmarks are summarized in Table 2. It is observed that our proposed method consistently improves the performance of student SNNs, enabling them to achieve significantly higher accuracy compared to existing training methods. Furthermore, we analyzed the test accuracy curves of directly trained SNNs and student SNNs guided by homogeneous teacher ANNs. As shown in Figure 3, learning from teacher ANNs enables student SNNs to increase their accuracy rapidly during training, especially on CIFAR100.

**Learning from the heterogeneous ANNs.** To demonstrate the effectiveness of knowledge transfer between heterogeneous teacher ANNs and student SNNs, we selected ResNet34, PyrNet110/50, and WRN28 as ANN teachers, aiming to improve the image classification performance of PyrNet50 and ResNet19 on CIFAR100 and Tiny ImageNet. The experimental results are shown in Table 3. The results show that student SNNs consistently achieve significant improvement when learning from heterogeneous ANNs, and in some cases even approach the teacher ANN’s performance. Specifically, on CIFAR100, the student PyrNet50 improves from a baseline SNN accuracy of 71.41% to 76.55% when taught by WRN28, which is very close to the teacher’s 76.60%. On Tiny ImageNet, the ResNet19 student achieves 55.35% accuracy when learning from PyrNet110, a substantial improvement over its baseline SNN accuracy of 51.51%. These results demonstrate that SAD-SNN enables effective cross-architecture knowledge transfer with only 2 time steps.

**Sensitivity Analysis of Hyperparameter α.** The balancing coefficient α in (6) controls the relative importance of the distillation loss versus the task loss. To evaluate the sensitivity of SAD-SNN to this hyperparameter, we conducted experiments on CIFAR100 using PyrNet110 and WRN28 as teachers and ResNet19 as the student, with α varying from 100 to 2000.

**Figure 4:**
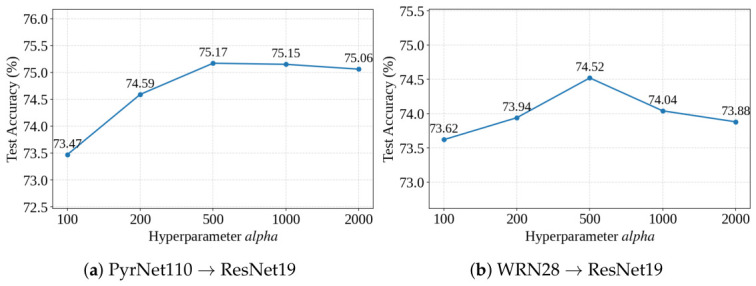
Hyperparameter sensitivity analysis of α in SAD-SNN on CIFAR100.

As shown in Figure 4, SAD-SNN achieves stable performance when α is in the range of 100 to 2000. When α is too small, the distillation signal is insufficient to guide the student SNN, resulting in performance close to the baseline without distillation (70.51%). When α is too large, the distillation loss dominates, potentially interfering with the task-specific learning and causing performance degradation. These results demonstrate that SAD-SNN is robust to α variations within a reasonable range.

#### 5.2.3. Theoretical Energy Consumption Calculation

We estimated the theoretical energy consumption of SNN-based image classification models following the methodology of prior works [45,46]. The computation starts from the synaptic operations (SOPs) in each layer. For layer *l*, the SOPs are calculated as:

(7) $$\mathrm{SOPs}\left(l\right)=r_{\mathrm{input}}\times T\times \mathrm{FLOPs}\left(l\right)$$

where $r_{\mathrm{input}}$ is the firing rate of the input spike sequence to layer *l*, *T* denotes the simulation time step, and FLOPs(l) is the number of floating-point multiply-accumulate (MAC) operations in that layer. The resulting SOPs(l) corresponds to the number of accumulate (AC) operations on spike signals.

Following the assumptions in [46], we adopted the energy costs for MAC and AC operations under a 45nm hardware architecture, where $E_{\mathrm{MAC}}=4.6\;\mathrm{pJ}$ and $E_{\mathrm{AC}}=0.9\;\mathrm{pJ}$. The total theoretical energy consumption of an SNN is then given by:

(8) $$E_{\mathrm{SNN}}=E_{\mathrm{MAC}}\times \mathrm{FLOPs}\left(1\right)+E_{\mathrm{AC}}\times \sum_{i=2}^{l}\mathrm{SOPs}\left(i\right)$$

where the first term FLOPs(1) accounts for the input layer, which encodes floating-point inputs into spike trains; the second term sums the AC energy over all subsequent layers. For comparison, the theoretical energy of an ANN model is simply:

(9) $$E_{\mathrm{ANN}}=E_{\mathrm{MAC}}\times \mathrm{FLOPs}$$

Table 3 reports the theoretical energy consumption on CIFAR100 and Tiny ImageNet (2 time steps), compared with the ANN baselines (PyrNet50 and VGG16).

#### 5.2.4. Performance Comparison of Distillation Knowledge

To investigate the effectiveness of the proposed Spatial-Activation Knowledge, we compared it against conventional logits-based knowledge distillation. We denote the method using logits-based distillation as Logits-based KD, and our proposed approach as Spatial-activation KD. The detailed results on three benchmark datasets are presented in Table 4. As shown, Spatial-activation KD consistently outperforms Logits-based KD across all teacher--student pairs on CIFAR10 and CIFAR100. Notably, the improvement is more obvious on the more challenging CIFAR100 dataset.

#### 5.2.5. Combination with Logits-Based Distillation

We empirically observe that SAD-SNN can be further combined with logits-based distillation to yield complementary gains. In order to better learn hidden knowledge in the teacher ANN, we introduce the temperature τ to make the logits distribution flatter. The output logits of both teacher ANN and student SNN are processed as follows:

(10) $$q_{i}^{t}=\frac{\exp(y_{i}^{t}/\tau )}{\sum_{j}\exp\left(y_{j}^{t}/\tau \right)};\;q_{i}^{s}=\frac{\exp(y_{i}^{s}/\tau )}{\sum_{j}\exp\left(y_{j}^{s}/\tau \right)}$$

where $y_{i}^{t}$ and $y_{i}^{s}$ denote the logits for class *i* from the teacher ANN and student SNN, respectively.

Following [22], we adopt the Kullback–Leibler divergence between the output distributions of the teacher and the student as the logits-based distillation loss.

(11) $$L_{distill\_logits}=\tau^{2}\cdot \mathrm{KL}\left(q^{t}\|q^{s}\right)=\tau^{2}\sum_{i}q_{i}^{t}\log\frac{q_{i}^{t}}{q_{i}^{s}}$$

Thus, the overall loss with the multi-level distillation loss can be expressed as follows:

(12) $$L_{all}=\gamma_{task}*L_{task}+\gamma_{SAD}*L_{distill\_SAD}+\gamma_{logits}*L_{distill\_logits}$$

where $\gamma_{task}$, $\gamma_{SAD}$, and $\gamma_{logits}$ are the hyperparameters controlling the weight of different losses.

In this study, we set the temperature τ to 4. Hyperparameters $\gamma_{task}$, $\gamma_{SAD}$, and $\gamma_{logits}$ were set to 0.5, 500, 0.5, respectively. Experimental results were shown in Table 5. It is obviously that multi-level distillation (Multi-levels KD) consistently outperforms SAD-SNN across all datasets. These results suggest that spatial-activation alignment captures complementary information to output-level supervision; the former enforces intermediate representation similarity, while the latter preserves semantic probability distributions. Notably, these improvements are consistent across both homogeneous and heterogeneous teacher settings.

### 5.3. Detection

#### 5.3.1. Implementation Details for Detection

**Datasets.** To evaluate the proposed method on electromagnetic signal detection, we construct a synthetic frequency-hopping signal dataset, denoted as RanFH. The generation pipeline consists of three stages: single-signal synthesis, time-frequency transformation, and annotation generation, all detailed in the Appendix A.

**Figure 5:**
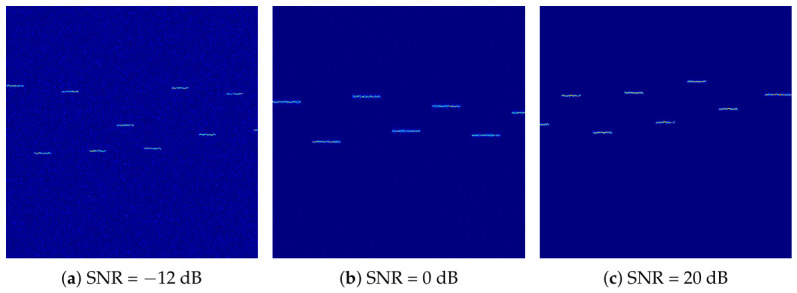
Example of RanFH data samples under different SNR conditions.

For the time-frequency transformation, signals are generated under SNR conditions ranging from −12 dB to 20 dB with a 2 dB step. Signals under each SNR are concatenated and transformed via STFT (window: 512, step: 256), extracting one image every 512 windows to yield 512 × 512 spectrograms. This process produces 4220 training images and 1028 validation images, subsequently resized to 640 × 640 × 3 with labels scaled accordingly. The detailed sample distribution across SNR levels is provided in Table A2. Figure 5 presents representative samples under three SNR conditions.

**Backbone Architectures.** In this study, we employed YOLOv5 Medium and YOLOv8 Medium [47] as the teacher ANNs to evaluate the transfer performance of SAD-SNN on the signal detection task, with SpikeYOLO [27] (24.44M parameters) serving as the student SNN. Detection performance is evaluated using *precision*, *recall*, and *mAP@*50:95 on the validation set. The detailed architectures of YOLOv5 Medium and YOLOv8 Medium are provided in Appendix B.4.

All experiments are conducted on a single NVIDIA A100 GPU with 80 GB memory under identical software and hardware conditions (same CUDA version, GPU, and a fixed random seed) to ensure fair comparison. For teacher ANNs, the number of epochs was set to 100, and the batch size was set to 8. We adopted the Adam optimizer with a single-cycle cosine annealing learning rate scheduler. The initial learning rate was 0.01 and decayed to 0.0001 over 100 epochs. During SNN training, the number of epochs was also set to 100, while the batch size was set to 16. The hyperparameter α in our proposed method was set to 1000 for all datasets. The time step was set to either 1 or 2. We employed the SGD optimizer with a single-cycle cosine annealing learning rate scheduler, where the learning rate was initialized at 0.01 and decayed to 0.0001 over 100 epochs.

#### 5.3.2. Main Results

For feature alignment, we map three feature layers of the teacher ANN to the corresponding layers of the student SNN. Specifically, for YOLOv8 Medium and SpikeYOLO, we align $L_{6}$, $L_{9}$, $L_{15}$ from the teacher with $L_{6}$, $L_{9}$, $L_{19}$ from the student; for YOLOv5 Medium and SpikeYOLO, we align $L_{6}$, $L_{9}$, $L_{17}$ with $L_{6}$, $L_{9}$, $L_{19}$. Among these, the first two aligned layer pairs correspond to features from the backbone, while the last pair corresponds to features from the head.

As shown in Table 6, transferring knowledge from the YOLOv8 Medium to the SpikeYOLO with two time steps, SAD-SNN achieves a mAP@50:95 of 92.67%, outperforming the directly trained SNN baseline. A similar trend is observed when using the YOLOv5 Medium, where SAD-SNN achieves 92.48% mAP@50:95, with a 1.68% improvement over the baseline. Notably, even with only one time step, our method consistently outperforms the baseline, achieving improvements of 0.38% and 0.24% for YOLOv8 Medium and YOLOv5 Medium, respectively. These results demonstrate that our SAD-SNN not only performs well on classification tasks but also exhibits strong generalization and effectiveness for electromagnetic signal detection, demonstrating that SAD-SNN is a general and effective solution for training high-performance SNNs with only two time steps.

#### 5.3.3. Inference Time and Theoretical Energy Analysis

We evaluate the inference time of SpikeYOLO models trained with different methods on a single NVIDIA A100 GPU with a batch size of 16, using 10 warm-up iterations followed by 50 test iterations. Following the setup in [27], the SOPs for SpikeYOLO with I-LIF neuron are calculated as

(13) $$\mathrm{SOPs}\left(l\right)=r_{\mathrm{input}}\left(l\right)\times T\times D\times \mathrm{FLOPs}\left(l\right)$$

where $r_{\mathrm{input}}\left(l\right)$ is the input spike firing rate, *T* is the time step, *D* is the I-LIF maximum integer value. The corresponding theoretical energy consumption is then obtained by applying the SOPs to (8).

We evaluated the inference time of SpikeYOLO models trained with different methods. The results are summarized in Table 6. It is observed that the inference time scales approximately linearly with the number of time steps. With a single time step, models trained with SAD-SNN achieve 7.84 ms/sample with YOLOv5 Medium and 8.07 ms/sample with YOLOv8 Medium. With two time steps, the inference time increases to 16.11 ms/sample and 16.09 ms/sample, respectively, approximately doubling that of the single-time step setting. Notably, the inference time of SAD-SNN-trained models is only marginally higher than that of the baseline, as our training strategy affects only the network weights and introduces no architectural modifications.

Table 6 also reports the theoretical energy consumption of SpikeYOLO models trained with different methods. It is observed that SAD-SNN-trained models consume 30.96 mJ with a single time step and 57.39 mJ with two time steps, compared to 26.38 mJ for the STBP baseline. The higher spike firing rate of SAD-SNN-trained models corresponds to increased theoretical energy consumption due to additional accumulate (AC) operations, while this is accompanied by consistent accuracy improvements. Notably, these are theoretical estimates based on the energy model under a 45nm hardware architecture, where MAC and AC operations consume 4.6pJ and 0.9pJ, respectively [48].

#### 5.3.4. Performance Under Varying SNR Conditions

**Figure 6:**
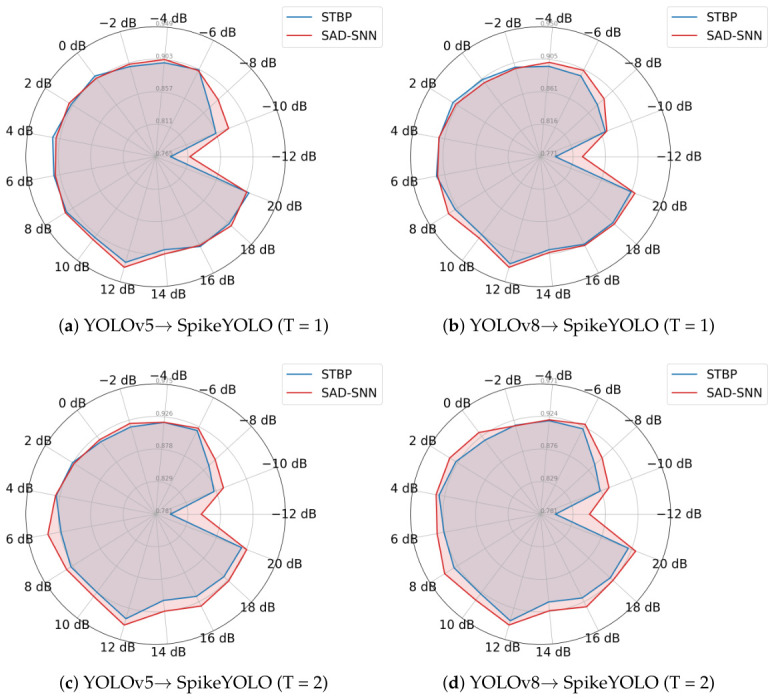
mAP50:95 of SpikeYOLO for each SNR in RanFH.

To evaluate the robustness of our method in practical scenarios, we analyzed detection performance across signal-to-noise ratio (SNR) levels ranging from −12 dB to 20 dB using radar plots, as shown in Figure 6. SAD-SNN consistently outperforms the STBP baseline across most SNR levels. The improvements are most evident under low-SNR conditions, where the performance gap between SAD-SNN and the baseline is largest. This indicates that the distilled knowledge helps the student SNN better recognize signal patterns even under heavy noise. As SNR increases, the gap narrows, as expected since the task becomes easier under cleaner conditions.

#### 5.3.5. Performance Comparison of Distillation Layers

To investigate the impact of different feature layer selections on distillation performance, we compare three alignment strategies: shallow-layer alignment ($L_{2}$, $L_{6}$, $L_{9}$), shallow-deep-layer alignment ($L_{6}$, $L_{9}$, $L_{17}$ for YOLOv5 Medium or $L_{6}$, $L_{9}$, $L_{15}$ for YOLOv8 Medium with $L_{6}$, $L_{9}$, $L_{19}$ of SpikeYOLO), and deep-layer alignment ($L_{17}$, $L_{20}$, $L_{23}$ for YOLOv5 Medium or $L_{15}$, $L_{18}$, $L_{21}$ for YOLOv8 Medium with $L_{19}$, $L_{22}$, $L_{25}$ of SpikeYOLO).

As shown in Table 7, shallow-deep-layer alignment achieves superior results: 92.48% for YOLOv5 Medium and 92.67% for YOLOv8 Medium under the same 2 × 4 setting. It is obvious that aligning intermediate feature layers—specifically, the last two backbone layers and the first head layer of the student with the corresponding layers of the teacher—provides the most effective knowledge transfer. Shallow layers lack sufficient semantic information, while overly deep layers suffer from task-specific specialization that may compromise generalizability.

## 6. Conclusions

In this work, we proposed a novel teacher–student learning approach named SAD-SNN to guide SNN training with comprehensive supervisory information from ANNs. SAD-SNN is a feature-based knowledge distillation method that preserves and transfers the spatial knowledge learned by ANNs to SNNs. Specifically, we introduce a spatial-activation loss with a normalization operation to align the representation vectors of ANN and SNN, alleviating the unexpected precision loss. To demonstrate the effectiveness of SAD-SNN, we selected ResNet19 and PyrNet50 as student SNNs for image classification, and SpikeYOLO as the student for electromagnetic signal detection. We compared SAD-SNN with existing SNN training methods on three image classification benchmarks and a signal detection dataset. Experimental results show that, regardless of whether homogeneous or heterogeneous teacher ANNs are used, the proposed SAD-SNN consistently outperforms other SNN training approaches using only two time steps across both tasks.

There are several promising directions for future research that are worth exploring. First, we see potential in applying SAD-SNN to enhance the performance of larger models, such as Spikeformer. This extension may promote the development of more complex and capable SNNs, pushing the boundaries of their applications. Second, we also have an interest in exploring methods to improve the performance of SNNs on neuromorphic datasets, such as DVS128 Gesture [49]. Furthermore, we intend to assess the reliability of SAD-SNN in more challenging, non-ideal environments, particularly focusing on its robustness to noise and interference in practical communication scenarios.

## Figures and Tables

**Table 1:** Summary of notations in this study.

Notation	Description
α	Balancing coefficient for distillation loss
*T*	Number of time steps
I	Set of aligned layer pairs $\left(l,l^{\prime }\right)$
$A_{T}^{l}\in R^{B\times C\times H\times W}$	Feature map of teacher ANN at layer *l*
$A_{S}^{l^{\prime }}\in R^{B\times T\times C^{\prime }\times H^{\prime }\times W^{\prime }}$	Feature map of student SNN at layer $l^{\prime }$
$A_{S^{\prime }}^{l^{\prime }}\in R^{B\times C^{\prime }\times H^{\prime }\times W^{\prime }}$	SNN feature map after average pooling over time dimension at layer $l^{\prime }$
$S_{T}^{l},S_{S^{\prime }}^{l^{\prime }}\in R^{B\times H\times W}$	Spatial-activation maps of teacher ANN and student SNN
$L_{\mathrm{task}}$	Task-specific loss
$L_{\text{distill\_SAD}}$	Spatial-Activation Distillation loss
$L_{\mathrm{logits}}$	Logits-based distillation loss
$L_{\mathrm{total}}$	Task loss with Spatial-Activation Distillation
$L_{\mathrm{all}}$	Task loss with both spatial-activation and logits distillation

**Table 2:** Top-1 accuracy (%) of SAD-SNN with existing methods on CIFAR10/CIFAR100. The best results are shown in boldface. Accuracy (%) of teacher ANNs: ResNet34/ResNet19: 95.32/94.56 on CIFAR10, 80.34/74.75 on CIFAR100; PyrNet110/PyrNet50: 95.74/95.61 on CIFAR10, 80.59/78.58 on CIFAR100. Accuracy (%) of student SNNs: ResNet19: 92.15/70.51 on CIFAR10/CIFAR100, PyrNet50: 92.60/71.41 on CIFAR10/CIFAR100.

Method	Training Categories	SNN	CIFAR10	CIFAR100	Time Step
Hybrid training [39]	Hybrid training	VGG11	92.22	67.87	125
Diet-SNN [40]	Hybrid training	ResNet-20	92.54	64.07	10/5
SNN_Calibration [41]	ANN-to-SNN	VGG16	93.71	77.68	32
Optimal_ANN-SNN [42]	ANN-to-SNN	VGG16	93.96	69.62	4
STBP [7]	Direct-training	CIFARNet	89.83	-	12
TSSL-BP [43]	Direct-training	CIFARNet	91.41	-	5
STBP-tdBN [33]	Direct-training	ResNet-19	92.92	70.86	4
TET [44]	Direct-training	ResNet-19	94.44	74.72	6
KDSNN [10]	KD training	ResNet-18	94.36	74.36	4
Reverse-KD [26]	KD training	ResNet-18	-	72.10	4
BKDSNN [11]	KD training	ResNet-19	94.52	74.76	4
SAD-SNN (ours)	KD training	ResNet34_ResNet19	**94.55**	74.94	2
		ResNet19_ResNet19	94.40	75.55	2
		PyrNet110_PyrNet50	93.45	**77.51**	2
		PyrNet50_PyrNet50	93.60	76.44	2

**Table 3:** Top-1 accuracy (%) and theoretical energy consumption (%) of *SAD-SNN* with 2 time steps on CIFAR100 and Tiny ImageNet. The down arrow (↓) indicates a reduction in energy consumption compared to the teacher ANN baseline.

	ANN-Model	SNN-Model	ANN	SNN	SOPs (G)	E (mJ)	SAD-SNN Acc
CIFAR100	PyrNet50	-	78.58	-	-	9.89	-
	ResNet34 WRN28	PyrNet50	80.34 76.60	71.41	1.33 1.36	1.20 (87.87% ↓) 1.23 (87.56% ↓)	77.64 76.55
	PyrNet110 WRN28	ResNet19	80.59 76.60	70.51	0.69 0.69	0.63 (93.63% ↓) 0.63 (93.63% ↓)	75.15 74.04
Tiny ImageNet	VGG16	-	56.10	-	-	5.77	-
	PyrNet110 PyrNet50	ResNet19	65.96 63.73	51.51	2.63 2.65	2.40 (58.41% ↓) 2.42 (58.06% ↓)	55.35 55.85

**Table 4:** Top-1 accuracy (%) of different distillation knowledge for SAD-SNN on three benchmarks.

	ANN-Model	SNN-Model	Logits-Based KD	Spatial-Activation KD
CIFAR10	ResNet34	ResNet19	92.85	94.55
	ResNet19	ResNet19	93.04	94.40
	PyrNet50	PyrNet50	93.51	93.60
CIFAR100	ResNet19	ResNet19	73.14	75.55
	PyrNet110	PyrNet50	76.60	77.51
	ResNet34	PyrNet50	76.29	77.64

**Table 5:** Comparison between SAD-SNN and multi-level knowledge distillation (Multi-levels KD) on three datasets.

	ANN-Model	SNN-Model	SAD-SNN	Multi-Levels KD
CIFAR10	ResNet34	ResNet19	94.55	94.66
	PyrNet110	PyrNet50	93.34	94.92
CIFAR100	WRN28	PyrNet50	76.55	77.44
	ResNet34	ResNet19	75.55	77.52
Tiny ImageNet	PyrNet110	ResNet19	55.35	61.14

**Table 6:** Detection performance, inference time, and theoretical energy consumption of SpikeYOLO with different training methods on the RanFH. T×D means that we set up *T* time steps, and each time step is expanded *D* times.

ANN Model	SNN Model	Training Methods	E (mJ)	T×D	Precision (%)	Recall (%)	mAP@50:95 (%)	Inference Time (ms/Sample)
YOLOv8	SpikeYOLO	STBP [7]	26.38	1 × 4	95.70	98.12	89.82	7.91
			49.71	2 × 4	96.16	97.59	90.80	15.52
		SAD-SNN	32.76	1 × 4	96.21	97.22	90.20	8.07
			57.39	2 × 4	96.37	97.05	92.67	16.09
YOLOv5	SpikeYOLO	STBP [7]	26.38	1 × 4	95.70	98.12	89.82	7.91
			49.71	2 × 4	96.16	97.59	90.80	15.52
		SAD-SNN	30.96	1 × 4	96.16	98.91	90.06	7.84
			56.27	2 × 4	96.24	97.58	92.48	16.11

**Table 7:** mAP@50.95 (%) of SAD-SNN for electromagnetic signal detection with different distillation layers. The best results are shown in boldface.

ANN Layers	SNN Layers	YOLOv5 → SpikeYOLO		YOLOv8 → SpikeYOLO	
		T×D **(1 × 4)**	T×D **(2 × 4)**	T×D **(1 × 4)**	T×D **(2 × 4)**
$L_{2}$, $L_{6}$, $L_{9}$	$L_{2}$, $L_{6}$, $L_{9}$	**90.08**	92.27	90.10	92.26
$L_{6}$, $L_{9}$, $L_{17}$	$L_{6}$, $L_{9}$, $L_{19}$	90.06	**92.48**	-	-
$L_{6}$, $L_{9}$, $L_{15}$	$L_{6}$, $L_{9}$, $L_{19}$	-	-	**90.20**	**92.67**
$L_{17}$, $L_{20}$, $L_{23}$	$L_{19}$, $L_{22}$, $L_{25}$	90.01	91.89	-	-
$L_{15}$, $L_{18}$, $L_{21}$	$L_{19}$, $L_{22}$, $L_{25}$	-	-	89.96	91.37

## Data Availability

All the used classification datasets are publicly available online. The synthetic RanFH dataset used for electromagnetic signal detection is available at https://github.com/fyzs1412/SAD-SNN.git (accessed on 27 July 2026).

## Appendix A. More Details About RanFH

### Appendix A.1. Single-Signal Generation

Each individual frequency-hopping signal is synthesized following a five-step procedure: (i) generation of random symbol sequences based on pseudo-random numbers; (ii) baseband modulation of the generated symbol sequences; (iii) application of a Hamming window to the modulated signals; (iv) frequency hopping implemented according to the same selection mechanism as the Link16 tactical data link; (v) injection of additive white Gaussian noise. The corresponding simulation parameters are summarized in Table A1.

**Table A1:** Simulation parameters of the RanFH dataset.

Parameter	Value
Sampling rate $f_{s}$ (Hz)	$500\times 10^{6}$
Start frequency $f_{0}$ (Hz)	$U(90\times 10^{6},180\times 10^{6})$
Symbol rate *v* (Hz)	$f_{s}/U\left(200,480\right)$
Hop rate (Hz)	v/U(24,48)
Hop duration (s)	$U\left(2000,4000\right)/f_{s}$
Modulation type	MSK, BPSK, QPSK
Hopping sequences per signal	U(8,12)
Min hop spacing (Hz)	$U(2\times 10^{6},4\times 10^{6})$
Hops per signal	U(12,24)

### Appendix A.2. Annotation Generation

Bounding box labels are produced concurrently with signal synthesis, recording for each hop the starting index, hopping frequency, and bandwidth, which is set to 1.2 times the symbol rate. These raw annotations are synchronously updated through the concatenation, STFT, and reshaping stages, with coordinates transformed accordingly. All annotations are finally exported in YOLO format for each image.

### Appendix A.3. Dataset Statistics

The detailed distribution of training and validation samples across the 17 SNR levels is summarized in Table A2. In total, the dataset comprises 4220 training images and 1028 validation images, with sample counts per SNR level ranging from 239 to 258 for training and 53 to 68 for validation.

**Table A2:** Distribution of training and validation samples across SNR levels in RanFH.

SNR (dB)	Train	Val	SNR (dB)	Train	Val
−12	246	63	4	250	62
−10	256	64	6	250	57
−8	242	65	8	243	64
−6	241	54	10	258	58
−4	256	53	12	239	60
−2	252	62	14	240	68
0	254	59	16	248	61
2	252	55	18	241	63
	20	252	60		

## Appendix B. Architecture Details

In our experiments, we employed six teacher ANN architectures for all datasets. For each classification dataset, the output dimension of the final fully connected layer was set to the corresponding number of classes: 10 for CIFAR10, 100 for CIFAR100, and 200 for Tiny ImageNet. For the electromagnetic signal detection task, we adopted YOLOv5n and YOLOv8s as teacher ANNs, with SNN-YOLOv8 as the student. The specific architectures for both tasks are detailed in the following subsections.

### Appendix B.1. ResNet Architectures

The architectural specifications for ResNet-19 and ResNet-34 are detailed in Table A3. Our implementation follows the standard ResNet [36] with the addition of a final linear layer as specified in ResNet-19 [17].

**Table A3:** Structure of ResNet-19 and ResNet-34.

Layer	Output Size		ResNet-19	ResNet-34
	CIFAR	Tiny ImageNet		
conv1	16×16	112×112	3×3, 64, stride 2	3×3, 64, stride 2
conv2_x	16×16	56×56	$\left[\begin{array}{c} 3\times 3,64\\ 3\times 3,64 \end{array}\right]\times 2$ (4 layers)	$\left[\begin{array}{c} 3\times 3,64\\ 3\times 3,64 \end{array}\right]\times 3$ (6 layers)
conv3_x	8×8	28×28	$\left[\begin{array}{c} 3\times 3,128\\ 3\times 3,128 \end{array}\right]\times 2$ (4 layers)	$\left[\begin{array}{c} 3\times 3,128\\ 3\times 3,128 \end{array}\right]\times 4$ (8 layers)
conv4_x	4×4	14×14	$\left[\begin{array}{c} 3\times 3,256\\ 3\times 3,256 \end{array}\right]\times 2$ (4 layers)	$\left[\begin{array}{c} 3\times 3,256\\ 3\times 3,256 \end{array}\right]\times 6$ (12 layers)
conv5_x	2×2	7×7	$\left[\begin{array}{c} 3\times 3,512\\ 3\times 3,512 \end{array}\right]\times 2$ (4 layers)	$\left[\begin{array}{c} 3\times 3,512\\ 3\times 3,512 \end{array}\right]\times 3$ (6 layers)
Global Avg Pool	1×1		AdaptiveAvgPool (1×1)	

### Appendix B.2. PyramidNet Architectures

We adopt PyramidNet with a widening factor $\alpha_{w}=270$. The network depth is controlled by a parameter *N* (either 50 or 110), where each of the three stages contains $N_{i}=\left(N-2\right)/3$ layers (i.e., 16 layers for PyramidNet-50 and 36 layers for PyramidNet-110). Note that PyramidNet maintains spatial resolution in the first stage (stride = 1) and performs downsampling at the beginning of subsequent stages. Table A4 presents the architectural configuration. The network utilizes additive pyramid-shaped feature dimension expansion controlled by the widening factor $\alpha_{w}$.

**Table A4:** Structure of PyramidNet-50 and PyramidNet-110.

Layer	CIFAR	Tiny ImageNet	PyramidNet-50	PyramidNet-110
conv1	32×32	64×64	3×3, 16, stride 1	3×3, 16, stride 1
conv2_x	32×32	64×64	$\left[\begin{array}{c} 3\times 3,16+\frac{\alpha_{w}\left(k-1\right)}{N}\\ 3\times 3,16+\frac{\alpha_{w}\left(k-1\right)}{N} \end{array}\right]\times 8$ (16 layers)	$\left[\begin{array}{c} 3\times 3,16+\frac{\alpha_{w}\left(k-1\right)}{N}\\ 3\times 3,16+\frac{\alpha_{w}\left(k-1\right)}{N} \end{array}\right]\times 18$ (36 layers)
conv3_x	16×16	32×32	$\left[\begin{array}{c} 3\times 3,16+\frac{\alpha_{w}\left(k-1\right)}{N}\\ 3\times 3,16+\frac{\alpha_{w}\left(k-1\right)}{N} \end{array}\right]\times 8$ (16 layers)	$\left[\begin{array}{c} 3\times 3,16+\frac{\alpha_{w}\left(k-1\right)}{N}\\ 3\times 3,16+\frac{\alpha_{w}\left(k-1\right)}{N} \end{array}\right]\times 18$ (36 layers)
conv4_x	8×8	16×16	$\left[\begin{array}{c} 3\times 3,16+\frac{\alpha_{w}\left(k-1\right)}{N}\\ 3\times 3,16+\frac{\alpha_{w}\left(k-1\right)}{N} \end{array}\right]\times 8$ (16 layers)	$\left[\begin{array}{c} 3\times 3,16+\frac{\alpha_{w}\left(k-1\right)}{N}\\ 3\times 3,16+\frac{\alpha_{w}\left(k-1\right)}{N} \end{array}\right]\times 18$ (36 layers)
Global Avg Pool	1×1		AdaptiveAvgPool (1×1)	

The factor *k* denotes the block index, *N* is the total number of blocks, and $\alpha_{w}$ is the widening factor.

The widening factor is set to $\alpha_{w}=270$, and *N* denotes the total network depth (N=50 for PyramidNet-50, N=110 for PyramidNet-110). The layer distribution parameters $N_{i}$ (where i∈{2,3,4}) are uniformly set to (N−2)/3 across all stages. The variable k∈[1,N+1] represents the current layer index, with channel dimensions increasing linearly from 16 to $16+\alpha_{w}$ throughout the network.

### Appendix B.3. WideResNet Architecture

The WideResNet architecture is characterized by a width multiplier *k* that expands the channel dimensions across all layers, as detailed in Table A5. For WideResNet-28, we set $N_{2}=N_{3}=N_{4}=n=4$, resulting in a total of 6n+4=28 layers (including convolutional and fully-connected layers).

**Table A5:** Structure of WideResNet-28.

Layer	Output Size	WideResNet-28	Layers
conv1	32×32	3×3, 16	1
block1 (conv2_x)	32×32	$\left[\begin{array}{c} 3\times 3,16k\\ 3\times 3,16k \end{array}\right]\times 4$	8
block2 (conv3_x)	16×16	$\left[\begin{array}{c} 3\times 3,32k\\ 3\times 3,32k \end{array}\right]\times 4$	8
block3 (conv4_x)	8×8	$\left[\begin{array}{c} 3\times 3,64k\\ 3\times 3,64k \end{array}\right]\times 4$	8
Global Avg Pool	1×1	AdaptiveAvgPool (1×1)	0

### Appendix B.4. YOLO Architectures

For the electromagnetic signal detection task, we employed YOLOv5 Medium and YOLOv8 Medium as teacher ANNs. Both models follow the standard Ultralytics YOLO design [47], consisting of a backbone for feature extraction and a detection head with three output scales (P3/8, P4/16, P5/32) to handle objects of varying sizes. The key difference lies in their building blocks: YOLOv5 Medium utilizes C3 modules, whereas YOLOv8 Medium adopts the more efficient C2f modules, which offer better gradient flow. Additionally, YOLOv8 Medium adopts an anchor-free design with a decoupled detection head, which separates the classification and regression branches to improve detection accuracy. The structural details are summarized in Table A6.

**Table A6:** Architectural comparison of YOLOv5 Medium and YOLOv8 Medium.

Component	Module	YOLOv5 Medium	YOLOv8 Medium
Backbone	Stem	Conv(64, 6, 2, 2) + Conv(128, 3, 2)	Conv(64, 3, 2) + Conv(128, 3, 2)
	Stage 1	C3(128) × 3	C2f(128, True) × 3
	Stage 2	Conv(256, 3, 2) + C3(256) × 6	Conv(256, 3, 2) + C2f(256, True) × 6
Backbone	Stage 3	Conv(512, 3, 2) + C3(512) × 9	Conv(512, 3, 2) + C2f(512, True) × 6
	Stage 4	Conv(1024, 3, 2) + C3(1024) × 3 + SPPF(1024, 5)	Conv(1024, 3, 2) + C2f(1024, True) × 3 + SPPF(1024, 5)
Head	Top-down	Conv(512, 1, 1) → Upsample → Concat(P4) → C3(512, False) × 3	Upsample → Concat(P4) → C2f(512) × 3
	P3 branch	Conv(256, 1, 1) → Upsample → Concat(P3) → C3(256, False) × 3	Upsample → Concat(P3) → C2f(256) × 3
	P4 branch	Conv(256, 3, 2) → Concat(P4) → C3(512, False) × 3	Conv(256, 3, 2) → Concat(P4) → C2f(512) × 3
	P5 branch	Conv(512, 3, 2) → Concat(P5) → C3(1024, False) × 3	Conv(512, 3, 2) → Concat(P5) → C2f(1024) × 3
Detection		Detect(P3, P4, P5) [nc = 1]	Detect(P3, P4, P5) [nc = 1]
Output	Feature Maps	P3/8 (80×80), P4/16 (40×40), P5/32 (20×20)	

Conv parameters are denoted as (channels, kernel size, stride, padding) where applicable. C3 and C2f are bottleneck modules from YOLOv5 and YOLOv8 respectively. SPPF denotes Spatial Pyramid Pooling Fast. P3, P4, P5 represent detection feature maps at 1/8, 1/16, and 1/32 scales.

## References

[1] McCulloch W.S., Pitts W. (1943). A Logical Calculus of the Ideas Immanent in Nervous Activity. Bull. Math. Biophys..

[2] Izhikevich E.M. (2003). Simple model of spiking neurons. IEEE Trans. Neural Netw..

[3] Stöckl C., Maass W. (2021). Optimized spiking neurons can classify images with high accuracy through temporal coding with two spikes. Nat. Mach. Intell..

[4] Zhang A., Cao H., Shan N., Wang J., Pu M., Song Y. (2025). Spiking neural networks for object detection and semantic segmentation across event-driven and frame-based modalities: A review. Intell. Opto-Electron..

[5] Lin C., Zhang Z., Wang L., Wang Y., Zhao J., Yang Z., Xiao X. (2024). Fast and lightweight automatic modulation recognition using spiking neural network. Proceedings of the 2024 IEEE International Symposium on Circuits and Systems (ISCAS), Singapore, 19–22 May 2024.

[6] Feng B., Zhu R., Zhu Y., Jin Y., Ju J. (2025). Dynamic vision sensor-driven spiking neural networks for low-power event-based tracking and recognition. Sensors.

[7] Wu Y., Deng L., Li G., Zhu J., Shi L. (2018). Spatio-Temporal Backpropagation for Training High-performance Spiking Neural Networks. Front. Neurosci..

[8] Shrestha S.B., Orchard G. (2018). Slayer: Spike layer error reassignment in time. Adv. Neural Inf. Process. Syst..

[9] Manohar V., Ghahremani P., Povey D., Khudanpur S. (2018). A teacher-student learning approach for unsupervised domain adaptation of sequence-trained asr models. Proceedings of the 2018 IEEE Spoken Language Technology Workshop (SLT), Athens, Greece, 18–21 December 2018.

[10] Xu Q., Li Y., Shen J., Liu J.K., Tang H., Pan G. (2023). Constructing deep spiking neural networks from artificial neural networks with knowledge distillation. Proceedings of the IEEE/CVF Conference on Computer Vision and Pattern Recognition.

[11] Xu Z., You K., Guo Q., Wang X., He Z. (2024). Bkdsnn: Enhancing the performance of learning-based spiking neural networks training with blurred knowledge distillation. Conference on Computer Vision.

[12] Rueckauer B., Lungu I.A., Hu Y., Pfeiffer M., Liu S.C. (2017). Conversion of continuous-Valued deep networks to efficient event-driven networks for image classification. Front. Neurosci..

[13] Cao Y., Chen Y., Khosla D. (2015). Spiking deep convolutional neural networks for energy-efficient object recognition. Int. J. Comput. Vis..

[14] Rueckauer B., Lungu I.A.H.Y., Pfeiffer M. (2016). Theory and Tools for the Conversion of Analog to Spiking Convolutional Neural Networks. arXiv.

[15] Yu Q., Gao J., Wei J., Li J., Tan K.C., Huang T. (2022). Improving Multispike Learning With Plastic Synaptic Delays. IEEE Trans. Neural Netw. Learn. Syst..

[16] Syed T., Kakani V., Cui X., Kim H. (2021). Exploring optimized spiking neural network architectures for classification tasks on embedded platforms. Sensors.

[17] Sengupta A., Ye Y., Wang R., Liu C., Roy K. (2019). Going Deeper in Spiking Neural Networks: VGG and Residual Architectures. Front. Neurosci..

[18] Voudaskas M., MacLean J.I., Dutton N.A., Stewart B.D., Gyongy I. (2025). Spiking neural networks in imaging: A review and case study. Sensors.

[19] Yang Z., Khairuddin A.S.M., Wong W.R., Chuah J.H., Noman H.M.F., Putri T.W.O. (2026). High-performance image classification via spiking vision transformer and an improved AA-LRSA attention mechanism. Neurocomputing.

[20] Middleton M., Ali T., Baikas E., Kayan H., Bhattacharya B.S., Gheorghiu E., Vousden M., Perera C., Rhodes O., Trefzer M.A. (2026). Event-based vision at the edge: A review. Brain Sci..

[21] Luo Z., Wang X.Y.S., Liu Z. (2024). Radar emitter recognition based on spiking neural networks. Remote Sens..

[22] Hinton G., Vinyals O., Dean J. (2015). Distilling the knowledge in a neural network. arXiv.

[23] Dong Y., Zhao D., Zeng Y. (2023). Temporal Knowledge Sharing enable Spiking Neural Network Learning from Past and Future. arXiv.

[24] Kundu S., Datta G., Pedram M., Beerel P.A. (2021). Spike-thrift: Towards energy-efficient deep spiking neural networks by limiting spiking activity via attention-guided compression. Proceedings of the IEEE/CVF Winter Conference on Applications of Computer Vision.

[25] Yu K., Yu C., Zhang T., Zhao X., Yang S., Wang H., Zhang Q., Xu Q. Temporal separation with entropy regularization for knowledge distillation in spiking neural networks. Proceedings of the Computer Vision and Pattern Recognition Conference.

[26] Xu Q., Li Y., Fang X., Shen J., Zhang Q., Pan G. (2024). Reversing structural pattern learning with biologically inspired knowledge distillation for spiking neural networks. Proceedings of the 32nd ACM International Conference on Multimedia.

[27] Luo X., Yao M., Chou Y., Xu B., Li G. (2024). Integer-valued training and spike-driven inference spiking neural network for high-performance and energy-efficient object detection. Proceedings of the European Conference on Computer Vision.

[28] Bengio Y., Courville A., Vincent P. (2013). Representation learning: A review and new perspectives. IEEE Trans. Pattern Anal. Mach. Intell..

[29] Romero A., Ballas N., Kahou S.E., Chassang A., Gatta C., Bengio Y. (2015). FitNets: Hints for Thin Deep Nets. arXiv.

[30] Gou J., Yu B., Maybank S.J., Tao D. (2021). Knowledge distillation: A survey. Int. J. Comput. Vis..

[31] Zagoruyko S., Komodakis N. (2016). Paying more attention to attention: Improving the performance of convolutional neural networks via attention transfer. arXiv.

[32] Neftci E.O., Mostafa H., Zenke F. (2019). Surrogate Gradient Learning in Spiking Neural Networks: Bringing the Power of Gradient-based optimization to spiking neural networks. IEEE Signal Process. Mag..

[33] Zheng H., Wu Y., Deng L., Hu Y., Li G. (2021). Going deeper with directly-trained larger spiking neural networks. Proc. AAAI Conf. Artif. Intell..

[34] LeCun Y., Bottou L. (1998). Gradient-based learning applied to document recognition. Proc. IEEE.

[35] Krizhevsky A., Hinton G. (2009). Learning multiple layers of features from tiny images. Handb. Syst. Autoimmune Dis..

[36] He K., Zhang X., Ren S., Sun J. Deep Residual Learning for Image Recognition. Proceedings of the IEEE Conference on Computer Vision and Pattern Recognition (CVPR).

[37] Han D., Kim J., Kim J. Deep pyramidal residual networks. Proceedings of the IEEE Conference on Computer Vision and Pattern Recognition.

[38] Zagoruyko S., Komodakis N. (2016). Wide residual networks. arXiv.

[39] Rathi N., Srinivasan G., Panda P., Roy K. (2020). Enabling deep spiking neural networks with hybrid conversion and spike timing dependent backpropagation. arXiv.

[40] Rathi N., Roy K. (2020). Diet-snn: Direct input encoding with leakage and threshold optimization in deep spiking neural networks. arXiv.

[41] Li Y., Deng S., Dong X., Gong R., Gu S. (2021). A free lunch from ANN: Towards efficient, accurate spiking neural networks calibration. Proceedings of the International Conference on Machine Learning, Virtual, 18–24 July 2021.

[42] Bu T., Fang W., Ding J., Dai P., Yu Z., Huang T. (2023). Optimal ANN-SNN conversion for high-accuracy and ultra-low-latency spiking neural networks. arXiv.

[43] Zhang W., Li P. (2020). Temporal spike sequence learning via backpropagation for deep spiking neural networks. Adv. Neural Inf. Process. Syst..

[44] Deng S., Li Y., Zhang S., Gu S. (2022). Temporal efficient training of spiking neural network via gradient re-weighting. arXiv.

[45] Hu Y., Deng L., Wu Y., Yao M., Li G. (2024). Advancing spiking neural networks toward deep residual learning. IEEE Trans. Neural Netw. Learn. Syst..

[46] Zhou Z., Zhu Y., He C., Wang Y., Yan S., Tian Y., Yuan L. (2022). Spikformer: When spiking neural network meets transformer. arXiv.

[47] Jocher G., Qiu J., Chaurasia A. Ultralytics YOLO. GitHub, 2023. https://github.com/ultralytics/ultralytics.

[48] Horowitz M. (2014). 1.1 computing’s energy problem (and what we can do about it). Proceedings of the 2014 IEEE International Solid-State Circuits Conference Digest of Technical Papers (ISSCC), San Francisco, CA, USA, 9–13 February 2014.

[49] Amir A., Taba B., Berg D., Melano T., McKinstry J., Di Nolfo C., Nayak T., Andreopoulos A., Garreau G., Mendoza M. A low power, fully event-based gesture recognition system. Proceedings of the IEEE Conference on Computer Vision and Pattern Recognition.

